# Unveiling the Potential: A Comprehensive Review of Artificial Intelligence Applications in Ophthalmology and Future Prospects

**DOI:** 10.7759/cureus.61826

**Published:** 2024-06-06

**Authors:** Uma Swaminathan, Sachin Daigavane

**Affiliations:** 1 Ophthalmology, Jawaharlal Nehru Medical College, Datta Meghe Institute of Higher Education and Research, Wardha, IND

**Keywords:** teleophthalmology, diabetic retinopathy, optical coherence tomography (oct), diagnostic accuracy, ophthalmology, artificial intelligence (ai)

## Abstract

Artificial intelligence (AI) has emerged as a transformative force in healthcare, particularly in the field of ophthalmology. This comprehensive review examines the current applications of AI in ophthalmology, highlighting its significant contributions to diagnostic accuracy, treatment efficacy, and patient care. AI technologies, such as deep learning algorithms, have demonstrated exceptional performance in the early detection and diagnosis of various eye conditions, including diabetic retinopathy (DR), age-related macular degeneration (AMD), and glaucoma. Additionally, AI has enhanced the analysis of ophthalmic imaging techniques like optical coherence tomography (OCT) and fundus photography, facilitating more precise disease monitoring and management. The review also explores AI's role in surgical assistance, predictive analytics, and personalized treatment plans, showcasing its potential to revolutionize clinical practice and improve patient outcomes. Despite these advancements, challenges such as data privacy, regulatory hurdles, and ethical considerations remain. The review underscores the need for continued research and collaboration among clinicians, researchers, technology developers, and policymakers to address these challenges and fully harness the potential of AI in improving eye health worldwide. By integrating AI with teleophthalmology and developing AI-driven wearable devices, the future of ophthalmic care promises enhanced accessibility, efficiency, and efficacy, ultimately reducing the global burden of visual impairment and blindness.

## Introduction and background

Ophthalmology plays a crucial role in healthcare, focusing on the diagnosis, treatment, and prevention of diseases and disorders related to the eye. With vision being one of the most valued senses, the field of ophthalmology is essential for maintaining overall health and quality of life [1]. Eye conditions, such as cataracts, glaucoma, diabetic retinopathy (DR), and age-related macular degeneration (AMD), are among the leading causes of vision impairment and blindness globally. As such, advancements in ophthalmic care have a profound impact on public health and well-being [2].

Artificial intelligence (AI) has emerged as a transformative force in healthcare, revolutionizing various aspects of medical practice, including diagnosis, treatment, and patient care. AI systems, powered by machine learning algorithms and deep neural networks, have demonstrated remarkable capabilities in analyzing complex medical data, detecting patterns, and making accurate predictions [3]. From image recognition to natural language processing, AI technologies are increasingly being integrated into clinical workflows to enhance efficiency, accuracy, and patient outcomes [4].

The purpose of this review is to examine the intersection of AI and ophthalmology, focusing on the current state of AI applications in the field and exploring future prospects. By synthesizing existing research and literature, this review aims to provide insights into how AI is being utilized to improve ophthalmic diagnosis, treatment, and patient care. Additionally, it seeks to identify challenges and opportunities in the adoption of AI technologies in ophthalmology and discuss their potential impact on the future of eye health.

## Review

AI in ophthalmic imaging

Automated Retinal Image Analysis for Early Detection of Diseases

Automated analysis of retinal images using AI holds significant promise for the early detection of various eye diseases. AI algorithms applied to retinal imaging techniques such as fundus photographs and optical coherence tomography (OCT) have shown remarkable accuracy in identifying conditions like AMD, DR, and other retinal diseases [5-7]. These AI systems can precisely segment retinal layers, categorize different retinal conditions, and forecast disease progression, thus facilitating the early detection of abnormalities and timely referrals for treatment [6]. The research highlights the potential of AI models like RETFound, which extract generalizable representations from unlabeled retinal images. These models can be adapted to various disease detection tasks using explicit labels, outperforming comparable models in diagnosing sight-threatening eye diseases and predicting systemic disorders with fewer labeled data [8]. Furthermore, AI systems have been devised to categorize multiple common referable fundus diseases and conditions, achieving efficiency and accuracy levels comparable to retina specialists. This capability enables swift diagnosis and treatment of patients, particularly in remote areas where specialized ophthalmologists are scarce [6]. The incorporation of AI into automated retinal image analysis provides a valuable tool for expanding screening initiatives, enhancing diagnostic precision, and facilitating early intervention for a wide spectrum of retinal diseases. These AI-driven systems not only streamline disease detection processes but also have the potential to transform the diagnosis and management of eye diseases, ultimately leading to better patient outcomes and improved access to timely care [9].

OCT Analysis for Diagnosis and Monitoring

OCT stands as a non-invasive imaging method extensively employed in ophthalmology to diagnose and monitor a variety of eye ailments. By utilizing light waves, OCT generates cross-sectional images of the retina, empowering ophthalmologists to visualize distinct retinal layers, gauge their thickness, and identify changes indicative of conditions such as glaucoma, AMD, and diabetic eye disease [10,11]. Moreover, OCT finds utility in diagnosing an array of retinal conditions, encompassing macular hole, macular pucker, vitreomacular traction, macular edema, retinal detachments, retinoschisis, and choroidal tumors [12]. It furnishes comprehensive insights into retinal structures, facilitating the discrimination of various eye disorders and aiding in devising treatment strategies. Additionally, OCT proves invaluable in monitoring glaucomatous structural damage involving the optic nerve, peripapillary retinal nerve fiber layer (RNFL), and macula, thereby enabling the evaluation of disease progression over time [10,13]. The evolution of OCT technology has heralded the advent of spectral-domain optical coherence tomography (SD-OCT) and swept-source optical coherence tomography (SS-OCT), which boast enhanced scan speeds, resolutions, and capabilities for imaging the eye's posterior segment with remarkable precision [10]. These advancements have significantly bolstered the diagnostic accuracy and management of eye conditions, positioning OCT as an indispensable tool in ophthalmic practice for diagnosing and monitoring diseases.

Advancements in Fundus Photography Interpretation Using AI

**Figure 1:**
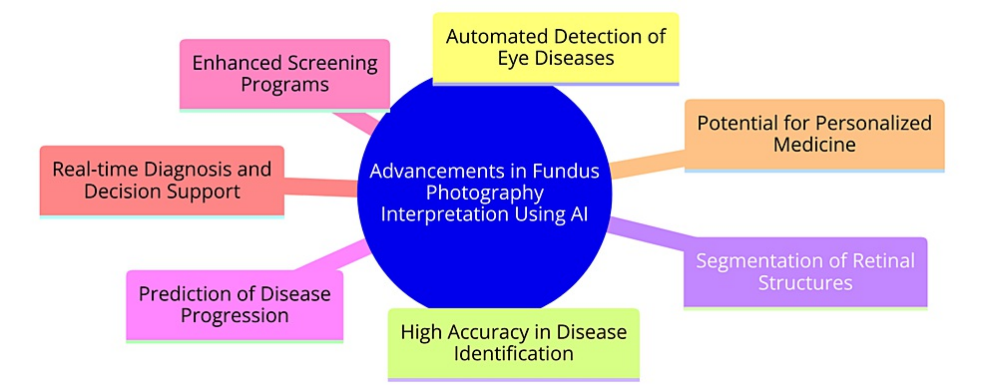
Advancements in fundus photography interpretation using AI Image credit: Dr Uma Swaminathan

AI systems have showcased remarkable proficiency in detecting and diagnosing eye diseases through fundus photographs [14,15]. These AI algorithms streamline the assessment of retinal conditions such as DR, AMD, and retinal vein occlusions (RVOs), consequently expediting diagnosis and referrals for positive cases [14,15]. AI-based fundus image analysis allows retinal vessels to be accurately segmented, arterioles and venules to be differentiated, and retinal geometric features to be quantified [15]. This capability enables the identification of systemic vascular disorders like hypertension and diabetes based on retinal microvascular abnormalities [15]. Deep learning algorithms have been devised to measure retinal vessel caliber from fundus photographs autonomously, leveraging previously measured ground truth data for training [15]. Moreover, AI models can directly classify systemic vascular disorders from fundus images without using specific feature extraction [15]. Implementing AI for interpreting fundus photography poses certain challenges, including the necessity for validated AI solutions with clinically acceptable performance, addressing issues related to image quality and patient population heterogeneity, instilling trust in AI-based systems, and standardizing reporting formats [16]. Despite these challenges, AI stands poised to revolutionize ophthalmology by augmenting disease detection, facilitating treatment planning, and improving patient care [16]. Advancements in fundus photography interpretation using AI are shown in Figure 1.

AI applications in ophthalmic diagnosis and prognosis

Automated Diagnosis of DR and AMD

AI is driving a paradigm shift in the automated diagnosis of DR and AMD, two primary causes of global vision impairment [17,18]. In DR, AI systems exhibit exceptional performance in discerning the disease from retinal images [17,19]. Notably, a study leveraging a deep learning algorithm achieved a sensitivity of 96.1% and specificity of 93.9% in detecting DR [19]. The FDA's approval of the first autonomous AI system (IDx-DR) for diagnosing DR in 2018 underscores the credibility and potential of AI in this domain [17,18]. These AI systems streamline the assessment of multiple eye scans, monitor disease progression, and facilitate timely referrals for positive cases [17,18]. Similarly, AI is making inroads in diagnosing AMD, a chronic and irreversible macular disease that ranks among the leading causes of central vision loss in the elderly [18]. While research on AI for AMD trails that of DR, burgeoning interest in the field has spurred several studies assessing the performance of AI grading systems in detecting various retinal diseases using retinal images [18]. The incorporation of AI into ophthalmic practices promises to significantly enhance patient outcomes and transform the diagnosis and management of eye diseases [17,20]. However, before AI systems can be widely adopted in clinical settings, security, privacy, and explainability must be diligently addressed [21]. Despite these hurdles, AI in ophthalmology is reshaping eye care practices, particularly in underserved regions with limited resources [21]. The automated screening and diagnosis capabilities of AI have the potential to mitigate the shortage of trained ophthalmologists and bolster access to essential eye care services [21].

AI-Assisted Glaucoma Diagnosis and Progression Tracking

AI is revolutionizing the diagnosis and progression tracking of glaucoma, enhancing the efficiency and accuracy of disease detection and management [22-24]. Across various imaging modalities such as OCT, fundus photography, and visual field (VF) testing, AI algorithms have exhibited outstanding performance in detecting glaucoma [22-24]. In diagnosing glaucoma, AI strategies analyze retinal photographs and synthesize risk factors to identify high-risk patients requiring further evaluation [22]. Deep learning techniques interpret OCT, VF testing, and other ocular imaging results to detect characteristic glaucomatous patterns [22-24]. Combining structural and functional inputs like OCT and VF data has significantly enhanced diagnostic accuracy, with area under receiver operating curve (AROC) values surpassing 0.90 [24]. Regarding glaucoma progression, AI algorithms have demonstrated the ability to detect progression earlier than conventional methods, potentially even from a single VF test [22-24]. AI-powered platforms facilitate continuous monitoring, with algorithms analyzing longitudinal data to alert physicians about rapid disease worsening promptly [22,23]. By integrating predictive analytics with patient-specific parameters, AI can guide precision medicine by aiding in individualized glaucoma treatment selections [22]. However, challenges, including external validity, explainability, and regulatory hurdles, must be addressed before widespread clinical implementation [22-24]. Ongoing longitudinal studies are imperative to assess AI interventions' long-term sustainability and efficacy in glaucoma care [22].

Predictive Analytics for Personalized Treatment Plans

Predictive analytics harnesses data, statistical algorithms, and machine learning to anticipate the likelihood of future outcomes based on historical data [25,26]. Within healthcare, this empowers providers to tailor treatment plans to individual patients rather than relying solely on population averages [25,26]. By scrutinizing patient data, including genetic information, lifestyle factors, and medical history, predictive analytics models can identify which treatments are most likely to be effective for a particular patient [27]. This personalized approach augments treatment efficacy and mitigates the risk of adverse events [26]. Furthermore, predictive analytics facilitates the identification of high-risk patient subgroups that stand to benefit from early interventions or preventive measures [25,26], ultimately fostering enhanced disease management and superior patient outcomes. Nevertheless, challenges persist in ensuring the generalizability of predictive models across diverse patient populations. The research indicates that models developed on one clinical dataset often exhibit diminished performance when applied to other datasets [28]. Addressing this challenge necessitates the cultivation of larger, higher-quality datasets, as well as the implementation of more stringent model validation procedures [28].

AI in ophthalmic surgery and intervention

Robot-Assisted Surgery in Ophthalmology

Robot-assisted surgery in ophthalmology incorporates robotic technologies to elevate surgical precision, control, and outcomes in eye procedures. These systems proffer advantages such as refined movement control, tremor cancellation, enhanced visualization, and distance sensing, effectively tackling challenges in tissue manipulation and intricate ocular surgeries [29]. An exemplary instance of such technology is the da Vinci Surgical System, which aids surgeons in executing intricate eye surgeries with remarkable accuracy and efficiency [29]. While robotics in ophthalmology are still in their nascent stages of development, advancements such as smart sensors and semi-autonomous surgery are anticipated to further augment surgical capabilities in the field [30]. The trajectory of robotic technology in ophthalmology holds tremendous potential for transforming surgical procedures, particularly in vitreoretinal and anterior segment applications, culminating in enhanced patient outcomes and diminished complications [29-31].

AI-Guided Laser Therapy for Retinal Diseases

AI-driven precision medicine has ushered in a transformative era in ophthalmology, particularly revolutionizing the process of fundus fluorescein angiography (FFA) imaging and diminishing the dependence on retinal specialists in FFA examination [32]. Deep learning systems (DLSs) have found widespread application in ophthalmology, where clinical practice relies on multimodal imaging for disease diagnosis and classification [32]. In retinal diseases such as retinal ischemia, AI-guided laser therapy is pivotal in prognosticating disease trajectory and visual outcomes for each patient. The identification and quantification of ischemia serve as foundational elements in guiding management principles and determining follow-up frequency for retinal vascular diseases, including RVO and DR [32]. AI systems excel in identifying ischemic eyes at elevated risk of complications, thereby facilitating timely interventions like laser photocoagulation when warranted [32]. Moreover, AI technologies are under development to furnish precise diagnosis and treatment recommendations for ischemic retinal diseases, offering a comprehensive approach spanning from diagnosis to treatment planning [32]. These strides in AI-guided laser therapy hold immense promise for enhancing patient outcomes, refining disease management, and optimizing the precision and efficacy of laser treatments for retinal diseases.

Virtual Reality (VR) and Augmented Reality (AR) Applications in Surgical Training and Planning

VR and AR applications have sparked a medical revolution, providing innovative solutions to enhance surgical education and elevate patient outcomes. These technologies furnish immersive simulated environments wherein trainees can practice surgical procedures devoid of risk, thereby fostering skill development and bolstering confidence [33,34]. In surgical training, AR and VR offer various benefits, including real-time feedback on trainees' performance, objective assessment of performance metrics, anatomical visualization, and exploration via 3D visualization of anatomy. Additionally, they facilitate remote mentoring and collaboration among surgeons globally [33,34]. By allowing surgeons to rehearse specific surgeries repetitively, refine their skills, and adapt to diverse scenarios before entering the operating room, AR and VR ultimately enhance surgical precision and improve patient safety [33,34]. Moreover, AR and VR streamline the integration of preoperative imaging, such as CT scans or MRIs, into the surgeon's field of view during procedures, enhancing precision and enabling personalized approaches tailored to the patient's unique anatomy [35]. Furthermore, these technologies facilitate holographic visualization of anatomy, aiding surgeons in navigating through intricate anatomical structures, executing precise incisions, and minimizing damage to surrounding tissues [35]. Additionally, AR and VR in surgical training and planning foster connectivity among remote clinical teams, enabling real-time collaboration, knowledge exchange, and expert consultations - an invaluable resource for training, skill enhancement, and seeking insights on complex cases [33,34]. Importantly, these technologies reduce radiation exposure during minimally invasive surgeries, mitigating health risks associated with repetitive ionizing radiation exposure for patients and medical staff [35].

Challenges and limitations

Data Privacy and Security Concerns

Ensuring robust data governance protocols is paramount in safeguarding sensitive health information within AI-driven ophthalmic practices. Encryption, access controls, and anonymization techniques should be rigorously implemented to mitigate risks associated with unauthorized access, data breaches, and potential misuse [36]. Continuous monitoring, auditing, and compliance verification are imperative for healthcare institutions to uphold the highest data privacy and security standards. These efforts ensure adherence to established protocols and regulations, fostering trust and confidence in AI-driven healthcare practices [36]. Transparency and informed consent are foundational principles in the ethical use of patient data for AI applications in ophthalmology. Clinicians and researchers must prioritize patient education, ensuring comprehensive understanding and informed consent regarding collecting, storing, and utilizing their health data. This approach empowers patients to make informed decisions about their participation in AI-driven initiatives, fostering trust and promoting ethical practices [36]. Balancing the imperative of data sharing to advance AI systems concerning patient privacy rights is essential. Ethical frameworks should oversee the entire data lifecycle, from acquisition to disposal, ensuring compliance with privacy regulations while advocating for responsible data-sharing practices. By striking this balance, healthcare organizations can foster collaboration and innovation while safeguarding patient privacy [36]. Addressing concerns over medical liability is crucial to fostering acceptance and adoption of AI systems in ophthalmology. Upholding ethical principles of transparency, accountability, and patient-centered care is essential. This ensures that the integration of AI in ophthalmology respects and protects patient privacy rights while harnessing the potential of data-driven innovations to improve clinical outcomes and quality of care [37,38]. Through proactive measures and adherence to ethical guidelines, healthcare professionals can navigate the complex landscape of AI-driven healthcare while prioritizing patient safety and well-being.

Regulatory Challenges in AI Adoption in Healthcare

The regulatory approval process for new AI-based medical technologies presents significant challenges, characterized by its sluggishness and complexity. Innovations can languish for years, navigating this process, hampering both adoption and innovation within the healthcare sector [39-41]. Additionally, the ambiguity surrounding liability in the event of AI system failures further complicates adoption. Healthcare providers are understandably cautious about embracing new AI technologies without clear guidelines on liability [39,41]. Privacy regulations, such as the General Data Protection Regulation (GDPR), hinder the effective collection and pooling of healthcare data necessary for training AI models. Heightened privacy concerns limit access to real patient data, impeding the development and refinement of AI-driven healthcare solutions [39,41]. Moreover, the absence of clear regulations tailored to the unique challenges posed by AI in healthcare exacerbates regulatory uncertainty. Existing frameworks were not designed to accommodate the rapid evolution of AI technologies, and defining precise regulatory requirements for abstract AI concepts remains a daunting task [39-41]. The distinction between adaptive and locked algorithms further complicates regulatory oversight. Regulators struggle to delineate when changes to AI algorithms necessitate re-review and re-authorization. While locked algorithms offer easier regulation, the innate desire of AI to learn and evolve over time presents a regulatory quandary [39,41]. Addressing bias in AI systems represents another significant challenge. Ensuring that AI systems are trained on high-quality, representative datasets to mitigate biases and discriminatory outcomes is paramount. However, the absence of official regulatory guidelines on training data usage exacerbates this challenge [39,41]. Furthermore, the lack of transparency in AI decision-making processes presents regulatory hurdles. Establishing transparency and explainability in AI algorithms is essential for fostering user trust. However, achieving this while navigating regulatory requirements remains a formidable challenge [39,41]. In sum, resolving these regulatory challenges is imperative for unlocking the full potential of AI in healthcare while ensuring patient safety, privacy, and fairness.

Ethical Considerations in AI-Assisted Diagnosis and Treatment

The ethical implications of AI in healthcare encompass various dimensions, including the risk of bias and discrimination. Inadequate or biased training datasets can inadvertently perpetuate biases and lead to unfair treatment of patients, underscoring the importance of proper design and monitoring of AI systems [42]. Moreover, ethical concerns arise regarding the validation and transparency of AI algorithms used for diagnosis or treatment. Robust validation processes and transparency in decision-making are essential for ensuring trust and accountability in medical decision-making [42]. Patient privacy and data governance represent critical ethical considerations in AI-assisted healthcare. Upholding patient privacy rights and ensuring meaningful control over data usage is imperative, necessitating clear communication and understanding of how patient data is utilized in AI systems [42]. Additionally, algorithmic fairness and addressing biases are paramount to preventing unjust outcomes and discrimination. Establishing guidelines and standards for reporting and comparing AI models can guide researchers and clinicians in developing ethical AI solutions [42]. While AI can enhance decision-making processes, maintaining human oversight and accountability is crucial. Clinicians must retain the ability to interpret and question AI outputs, recognizing the limitations of AI systems and ensuring that human judgment remains integral to the diagnostic and treatment process [42,43]. Furthermore, developing comprehensive ethical frameworks and governance mechanisms is essential for the responsible integration of AI technologies in medical practice. Prioritizing patient safety, fairness, and ethical compliance is imperative to ensure the ethical use of AI in healthcare settings [42,43].

Future prospects and emerging trends

Integration of AI With Teleophthalmology for Remote Patient Care

AI-enabled teleophthalmology is revolutionizing access to eye care, particularly for underserved and remote populations. By leveraging AI, patients in such areas can now access vital eye care services through remote monitoring and diagnosis, addressing critical conditions like DR and retinopathy of prematurity [44,45]. Moreover, AI systems enhance disease screening and diagnosis by swiftly analyzing retinal images captured via smartphone-based devices or wearable head-mounted cameras. This innovation enables remote screening and diagnosis of eye diseases, such as DR, with remarkable accuracy [45,46]. The advent of teleophthalmology, facilitated by AI, is significantly reducing the need for in-person visits, particularly for follow-up care. Through remote monitoring, patients can conveniently manage their eye health. At the same time, easy-to-use devices like at-home OCT scanners empower individuals to capture images for remote interpretation by ophthalmologists [47]. Additionally, integrating AI with teleophthalmology holds the potential for earlier intervention in eye diseases. By facilitating earlier detection, AI enables timely referrals and interventions, ultimately improving patient outcomes and mitigating disease progression [46].

Development of AI-Driven Wearable Devices for Continuous Monitoring of Eye Health

The emergence of AI-driven wearable devices, designed for continuous eye health monitoring, represents a cutting-edge research frontier with immense potential to transform the accessibility and management of eye care. These groundbreaking devices, ranging from smart eyewear to smart contact lenses, are equipped with advanced sensors, optical displays, and AI capabilities, offering many applications in ophthalmology care [48,49]. AI-driven wearable devices can gather comprehensive, continuous, and individual-specific data, a feat often challenging to achieve in traditional clinical settings. By harnessing sophisticated data processing and AI algorithms, these devices can pinpoint at-risk patients, detect behavioral patterns, and facilitate timely interventions for eye-related conditions [48,49]. These devices hold promise for providing real-time monitoring, enabling remote assessments, facilitating telehealth consultations, and delivering personalized interventions, thereby enhancing the overall management of vision impairments and eye diseases [48,49]. Furthermore, AI algorithms embedded within these wearable devices can analyze extensive physiological and biometric data, identifying patterns and anomalies that may escape human detection. This capability enables early detection of eye diseases, anticipation of potential health issues, and proactive interventions, ultimately culminating in improved patient outcomes and tailored treatment approaches [48,49].

AI-Powered Drug Discovery for Ophthalmic Conditions

AI-powered drug discovery for ophthalmic conditions is a cutting-edge area of research that leverages AI technologies to revolutionize the development of pharmaceutical products for eye diseases. By analyzing large datasets and identifying potential drug targets, AI algorithms play a crucial role in predicting drug efficacy and toxicity, streamlining clinical trial design, and enhancing the overall efficiency and accuracy of drug development processes in ophthalmology [50]. These AI-driven approaches enable researchers and clinicians to gain deeper insights into glaucoma-related lesions, predict disease progression, and differentiate between healthy and affected eyes, ultimately leading to more precise and effective treatment strategies for ophthalmic conditions [50]. The integration of AI in drug discovery for ophthalmic conditions marks a significant advancement in the field, offering the potential to accelerate the identification of novel therapeutic targets, optimize treatment regimens, and improve patient outcomes. By harnessing the power of AI, researchers can enhance their understanding of eye diseases, predict treatment responses, and tailor interventions to individual patient needs, ushering in a new era of personalized medicine in ophthalmology [50].

## Conclusions

In conclusion, AI has substantially contributed to ophthalmology, significantly enhancing diagnostic accuracy, treatment precision, and patient care. AI technologies, particularly those involving deep learning algorithms, have shown remarkable efficacy in detecting and diagnosing a range of eye conditions, such as DR, glaucoma, and AMD. These advancements enable earlier and more accurate diagnoses, improving patient outcomes. Additionally, AI's integration into surgical assistance and predictive analytics promises to elevate clinical efficiency and personalize treatment plans. The transformative potential of AI in ophthalmology extends to expanding access to eye care through teleophthalmology and pioneering new treatments via AI-driven drug discovery. However, realizing the full potential of AI in this field requires ongoing research, collaboration, and investment to address challenges related to data privacy, regulatory compliance, and ethical considerations. By fostering partnerships among clinicians, researchers, technology developers, and policymakers, the global healthcare community can leverage AI to advance eye health and reduce the incidence of visual impairment worldwide.
